# The Global Impact of Sepsis: Epidemiology, Recognition, Management, and Health System Challenges

**DOI:** 10.3390/epidemiologia7010020

**Published:** 2026-02-03

**Authors:** Luigi La Via, Salvatore Ferlito, Maria Stella Di Modica, Andrea Marino, Giuseppe Nunnari, Bruno Cacopardo, Jerome Rene Lechien, Mario Lentini, Salvatore Lavalle, Giancarlo Carmelo Botto, Paolo Buscema, Loris Gruppuso, Antonino Maniaci

**Affiliations:** 1 Department of Anesthesia and Intensive Care, University Hospital Policlinico “G. Rodolico-San Marco”, 24046 Catania, Italy; 2 Department of Medical, Surgical Sciences and Advanced Technologies “GF Ingrassia” ENT Section, University of Catania, 95123 Catania, Italy; 3 Unit of Infectious Diseases, Department of Clinical and Experimental Medicine, ARNAS Garibaldi Hospital, University of Catania, 95122 Catania, Italy; mariastella.dimodica@unikorestudent.it (M.S.D.M.); salvatore.lavalle@unikore.it (S.L.); 4 Department of Medicine and Surgery, University of Enna “Kore”, 94100 Enna, Italy; andrea.marino@unict.it (A.M.); cacopard@unict.it (B.C.); 5 Department of Surgery, UMONS Research Institute for Health Sciences and Technology, University of Mons, 7000 Mons, Belgium; 6 Asp 7 Ragusa, Ospedale Maggiore Modica, 97015 Ragusa, Italy

**Keywords:** sepsis epidemiology, early recognition, rapid diagnostics, antimicrobial stewardship, health systems strengthening

## Abstract

Background: Sepsis constitutes a major healthcare burden worldwide, with an estimated 48.9 million incident cases and 11.0 million deaths in 2017, accounting for nearly one-fifth of all global deaths. Even with advances in definitions and guidelines, significant inequalities persist in awareness, early treatment, and health system readiness. Methods: We performed a structured narrative review of epidemiology studies, clinical case definitions, diagnostic approaches, stewardship interventions, and health system reports. Both electronic sources (PubMed, Web of Science, Embase, Scopus) and grey literature (World Health Organization [WHO], National Institute for Health and Care Excellence [NICE], Society Critical Care [SSC]) were explored. Evidence incorporated themes were organized across recognition, diagnostics, antimicrobial therapy, organ support, guidelines, and health system determinants. Results: Measurement tools, including quick Sequential Organ Failure Assessment (qSOFA) and Sequential Organ Failure Assessment (SOFA), exhibited suboptimal sensitivity and utility in varied clinical environments. Biomarkers (procalcitonin, presepsin, CD64) and rapid molecular diagnostics, including metagenomic next-generation sequencing (mNGS) and AI-based devices, enhance detection but are limited by cost and infrastructure constraints. Each hour of delay in antibiotic therapy is associated with a 6–10% increased risk of mortality, underscoring the importance of stewardship, including the incorporation of empiric regimens with rapid de-escalation. Health system bottlenecks—human resources, funding, infrastructure—continue to be a significant determinant of outcomes, especially in low- and middle-income countries. Conclusions: Attaining the 2030 WHO targets for sepsis involves precision diagnostics, adaptable guidelines, stewardship frameworks, and resilient health systems. Fair application and resource allocation are crucial to lower the incidence and mortality worldwide.

## 1. Introduction

Sepsis, which is characterized by the Sepsis-3 consensus statement as “life-threatening organ dysfunction due to a dysregulated host response to infection” [1], continues to be a significant worldwide health problem. This transition from phenotypic definitions achievable with Systemic Inflammatory Response Syndrome (SIRS) to organ dysfunction as defined by the Organ Failure Assessment Score (SOFA) criteria has fundamentally altered diagnostic and epidemiological spaces [1,2]. However, this semantic drift has also created difficulties in the comparison of historical with present-day data, potentially masking trends in the incidence and outcomes by geographic areas [3].

Sepsis continues to represent a major global health challenge, with substantial geographic variability in incidence and mortality linked to healthcare disparities and access to early intervention [4,5,6,7]. Despite age-adjusted incidence and mortality rates decreasing by approximately one-third and one-half, respectively, from 1990 to 2017, the absolute burden is large because of population growth and ageing [4].

According to the Global Burden of Disease (GBD) Study 2017, sepsis accounted for 48.9 million cases and 11.0 million deaths, representing 19.7% of all global deaths, despite age-adjusted declines since 1990 [2].

Sepsis-associated in-hospital mortality in high-income countries is 15–25% and up to 30–40% in septic shock [6]. These numbers highlight the significant morbidity and mortality that continue to occur despite improvement in critical care.

The epidemiological load of sepsis becomes apparent notably in certain risk groups. Almost 2.9 million annual sepsis deaths occur in children under five, and maternal sepsis still accounts for around 10.7% of global maternal deaths [7,8]. Low- and middle-income countries are most severely affected, particularly in sub-Saharan Africa, South Asia and Oceania, where poor health systems and limited access to early observation and treatment would all contribute to increased risks [4,7]. In contrast, high-income countries (HICs) report lower case fatality rates due to better access to intensive care and early sepsis recognition programmes. This disparity underscores how regional disparities are at the core of the global sepsis burden. Recent investigations further highlight the persistence of global heterogeneity in sepsis outcomes despite major advances in critical care. Large-scale multicountry analyses published between 2023 and 2025 have shown that sepsis-related mortality remains high, particularly in low-resource regions where delayed recognition and limited access to organ support are prevalent [7,9,10,11]. New epidemiological models have refined estimates of disease burden by incorporating non-hospital and community-onset cases, revealing that true incidence may exceed earlier Global Burden of Disease projections [2,9]. In parallel, recent studies have emphasized the role of diagnostic innovations—such as rapid molecular testing, artificial intelligence-based triage, and biomarker-guided decision tools—in improving early detection and management, yet their adoption remains inconsistent worldwide [10,11]. These updated data reinforce the need for integrated approaches that address both clinical and systemic barriers to effective sepsis care.

However, sepsis continues to be underdiagnosed in both the clinical environment and the public domain. Estimates of how frequently sepsis occurs are imprecise, and mortality rates differ between countries; however, in China, Klebsiella pneumoniae has been identified as one of the most frequent bacterial pathogens responsible for sepsis, particularly in hospital-acquired and intensive care unit (ICU) settings [7]. Although public awareness of sepsis is low and it is a potentially fatal condition, a US survey found that 81% of the public did not know what sepsis was, and a significant number of cases develop in the community setting, where the opportunity to detect it early is greatest [12]. This lack of knowledge may lead to delayed presentation and diagnosis, with poor outcomes. The changing concept of sepsis has also had implications for epidemiologic surveillance. While the Sepsis-3 criteria focus on organ dysfunction measured by SOFA or qSOFA [2], subsequent studies have noted variable diagnostic performance depending on resource availability and patient population characteristics [13,14]. In addition, hospitals and quality improvement programmes, such as those in the Centers for Disease Control and Prevention (CDC) Adult Sepsis Event criteria or the Surviving Sepsis Campaign guidelines, use different methods to operationalize diagnosis, making comparisons difficult [9].

These definitional and operational differences present significant challenges for global benchmarks and policy formulation. For example, the new Sepsis-3 criteria have resulted in a higher estimated number of patients identified as septic in some places, raising comparison issues with existing estimates based on SIRS and highlighting how essential standardized surveillance systems are [3].

The sequelae of sepsis are, however, not limited to the acute mortality. In patients, the impact has often been characterized by large reductions in quality of life, increased cognitive impairment, and increased post-discharge mortality, particularly in the elderly or those with pre-existing chronic disease [7]. These downstream consequences magnify the total effect, magnifying the costs, which are again, not just healthcare costs, but all social and economic costs.

Sepsis, therefore, represents a compelling global public health issue. Its changing definitions, enormous epidemiological dimensions, and profound health system challenges—from loss of recognition and treatment to lack of comparability—are a call for an overarching synthesis of current evidence. This review seeks to scrutinize major elements of sepsis, including global impact, epidemiology, clinical management strategies, and whole-body responses. Through combining quantitative estimates, diagnostic frameworks and policy context, we aim to highlight key lacunae and potential opportunities to shape future research, raise the profile and invigorate the global apparatus for sepsis prevention, detection and management.

The main objective of this review was to provide a comprehensive synthesis of current global evidence on the epidemiology, recognition, and management of sepsis, with a focus on system-level challenges that affect outcomes. Specifically, we aimed to (1) summarize contemporary epidemiological trends and identify geographic disparities; (2) evaluate the performance and applicability of diagnostic and prognostic tools; (3) analyze evidence-based strategies for timely treatment, antimicrobial stewardship, and organ support; and (4) discuss health system barriers and policy frameworks influencing sepsis care. We hypothesize that persistent global inequalities in recognition and management contribute substantially to sepsis-related mortality, and that integrating stewardship, precision diagnostics, and adaptive health system responses can mitigate these disparities.

Ultimately, this review captures these observations within Key Conceptual Messages that consolidate what diagnostics, stewardship, systems-based constraints and emerging technologies mean in varying resource contexts. These messages, reported in the Conclusion section, explicate the system-level implications of our synthesis and demonstrate how this review carries earlier studies forward.

## 2. Materials and Methods

### 2.1. Search Strategy and Data Sources

We designed a comprehensive review to integrate and critically evaluate the current global burden of sepsis, focusing on its epidemiology, challenges in bedside management, as well as systemic issues. We searched PubMed/MEDLINE, Embase, Web of Science and Scopus to identify eligible studies published.

Our literature search included articles that were last updated in November 2025 to ensure inclusion of the most recent peer-reviewed studies available at the time of manuscript preparation. Search terms included *sepsis, septic shock, epidemiology, health systems, global burden, sepsis guidelines, sepsis management and their combination with barriers (and) diagnostics (or) antimicrobial stewardship; Sepsis-3; WHO policy*. The search was optimized for sensitivity and specificity using Boolean operators and MeSH terms. Manual citation tracking and grey literature searches (World Health Organization reports, Global Sepsis Alliance documents, conference abstracts) were also conducted to identify non-indexed items.

We followed the SANRA (Scale for the Assessment of Narrative Review Articles) to inform its construction, structure and presentation, in particular concerning transparency of search strategy, referencing completeness, scientific rationale and coherence of argumentation. SANRA was used exclusively as a methodological approach for structuring the process of the review and not as an approach to assess quality or risk of bias at the individual primary study level. Due to the diverse scope of included literature (epidemiologic modelling studies, diagnostic accuracy investigations, stewardship interventions, and policy documents), we did not use a formal risk-of-bias tool (ROBIS, QUADAS-2 or GRADE) at the study level. This choice is recognized as a methodological limitation.

#### 2.1.1. Sampling and Data Validation Procedures

We imported all retrieved records into a reference management system (Zotero 6.0) to remove duplicates before screening. Two independent reviewers (A.M. and M.S.D.M.) screened titles and abstracts using predefined inclusion and exclusion criteria. A full-text review was subsequently performed to confirm eligibility, and disagreements were resolved through discussion with a third reviewer (S.F.). Sampling was designed to ensure comprehensive representation across geographic regions, healthcare income levels, and study types (epidemiological, clinical, and policy-based). We achieved data validation by cross-checking extracted information from each included study (population characteristics, definitions used, outcomes, and quality indicators) with at least one other independent source or guideline (e.g., WHO, SSC, NICE). Where we identified discrepancies, consensus decisions were documented in the extraction matrix. We adopted the SANRA (Scale for the Assessment of Narrative Review Articles) checklist to ensure study quality and reporting completeness by methodological consistency and transparency. Each included study was assessed in terms of the SANRA items: (1) relevance, (2) search strategy and sources used, (3) referencing, (4) scientific reasoning and interpretation of results, (5) evidence level support for conclusions drawn and (6) discussion about limitations. This is a structured method to optimally evaluate and summarize included evidence while avoiding binary scoring used for systematic review designs.

#### 2.1.2. Critical Appraisal and Quality of the Evidence

This review includes recent data on epidemiology, diagnosis and clinical management, but the studies are heterogeneous in terms of methodology. Most epidemiologic estimates, including Global Burden of Disease (GBD) models and WHO reports, are based on modelling methods with insufficient LMIC data available to inform the models, and likely underestimate disease burden. Meta-analyses assessing recognition instruments (qSOFA, SIRS, NEWS2) frequently include heterogeneous patient cohorts and various sepsis definitions with diverse time points of measurement of the score; thus, results are barely comparable, and transferability is limited. Biomarker trials, especially of presepsin and CD64, suffer from arbitrary cut-off values, single-centre derivation cohorts and spectrum bias. In addition, many of the rapid diagnostic trials (mNGS) do not have randomization comparison and are in high-resource settings where not all resources are available in LMICs. Recommendations from guidelines (SSC, NICE, WHO) are also based on evidence of uneven quality; some treatment components rely upon observational data or expert opinion rather than large RCTs and there are substantial inconsistencies concerning the optimal amounts of fluid administration for resuscitation, timing of RRT initiation and supplemental therapies like steroids or Vit C. By explicitly addressing these limitations our synthesis aspires to situate strength of evidence and delineate the areas in which caution and local adaptation may still be appropriate.

### 2.2. Eligibility Criteria and Search Strategy

Only peer-reviewed studies, clinical guidelines, policy documents, and global health reports that fulfilled the following criteria were included as follows:

- Human sepsis or septic shock in adult pediatric populations;
- Included epidemiological studies, clinical definitions or diagnostic strategies, treatment strategies or health system implementation;
- Global, regional, or national in extent;
- Were written in English, French or Spanish.

We also excluded animal studies, laboratory-only models, and opinion pieces without data points or any structured opinion. Articles were reviewed by two independent investigators (AM, MSDM) with the use of title/abstract and full text. Any discrepancies were resolved by agreement or consultation with a third assessor. The data included variables such as region, population studied, study design, outcome measures and relevance to 1 of 9 predefined thematic domains; the key data for each question were extracted in standard matrix-formatted sheets. To integrate findings, a narrative synthesis approach was used and structured following the paragraph-based thematic framework of the review.

### 2.3. Statistical and Analytical Approach

As this review was designed as a structured narrative synthesis, quantitative data were summarized descriptively rather than through formal meta-analytical pooling. We extracted numerical values (incidence, prevalence, mortality rates, and diagnostic performance metrics) and verified against primary sources and official repositories (WHO, Global Burden of Disease, and CDC datasets). We reported central tendencies and ranges directly as cited in the original studies. When multiple studies reported overlapping data, we prioritized the most recent or methodologically robust estimate. Data consistency was verified by comparing extracted figures across different databases and recalculating proportions when necessary. Statistical assumptions (such as homogeneity of estimates or overlapping confidence intervals) were qualitatively assessed to avoid misinterpretation of heterogeneous datasets. All graphical elements were generated using aggregated descriptive data only, ensuring transparency and reproducibility of the synthesis.

### 2.4. Overview of the Quality of Evidence Across Domains

Evidence included in this review comes from heterogeneous designs with varying methodological quality across the domains. Most epidemiological estimations are based on modelling studies, national administrative databases and multicountry retrospective analyses with very limited prospective surveillance reports from LMICs. Available evidence for the clinical scoring systems (qSOFA, SIRS, NEWS2) is mainly derived from retrospective cohort studies and meta-analyses with heterogeneous patient populations, as well as variation in measurement time points and definitions of outcome. Many of these investigations, for PCT as well as for presepsin and CD64, are single-centre or in high-resource settings only and have used non-standardized assays with significant variability between different reports. The evidence for quality-improvement programmes, antimicrobial stewardship, and system-level interventions is based largely on observational or quasi-experimental studies rather than randomized trials or prospective multicenter evaluations. This heterogeneity in quality of evidence should inform cautious interpretation of pooled estimates and comparisons across contexts.

## 3. Results

### 3.1. Global Epidemiology of Sepsis

Building on prior global estimates, recent epidemiological analyses have refined our understanding of regional incidence, mortality, and temporal trends in sepsis.

Much of the global incidence data is model-derived rather than directly measured, and data scarcity in LMICs introduces substantial uncertainty, leading to wide credible intervals.

These epidemiologic disparities reinforce Proposition 5, indicating that health system capacity—not pathogen biology—is the dominant determinant of sepsis mortality across regions.

#### 3.1.1. Incidence and Prevalence: Relative Regional and Temporal Trends

**Figure 1:**
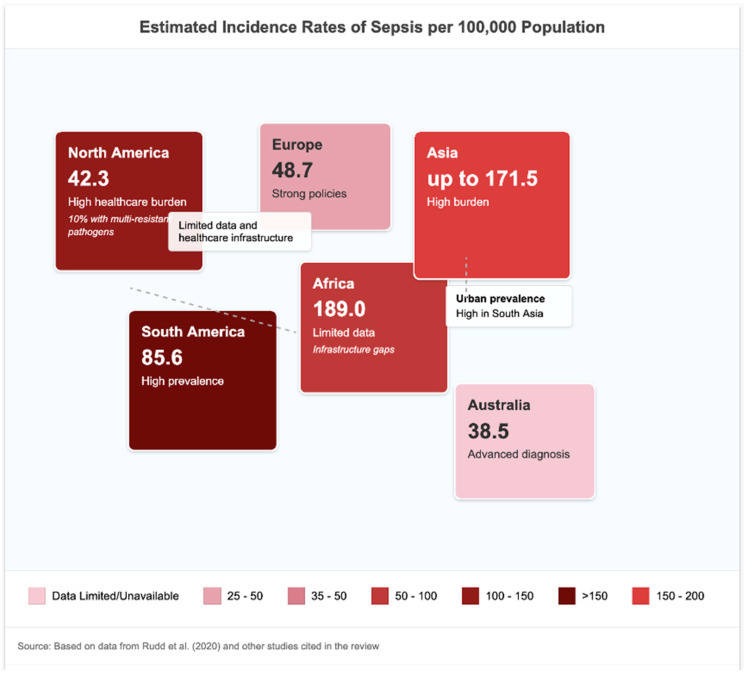
Estimated global prevalence of sepsis by world region (cases per 100,000 population per year), generated from aggregated data reported by WHO (2020) [4], the Global Burden of Disease Study (2020) [2], and Fleischmann-Struzek et al. (2020) [9].

The global burden of sepsis is large and unequal. Recent estimates from analyses of meta-analyses and modelling studies, based mainly on healthcare data from high-income countries, propose annual global incidence rates, varying between 276 and 678 cases per 100,000 persons [13]. Global-level transmission model scenarios estimated that the world endured ~48.9 million cases and 11.0 million deaths in 2017, representing approximately 19.7% of global deaths, according to the Global Burden of Disease (GBD) Study 2017 [2,14]. Concomitant data from the WHO support these data and highlight that around 50% of the cases are in children under 5 years of age [9] (Figure 1).

In extensive meta-analyses and GBD data, the pooled global incidence per 100,000 persons ranged across 437 (95% CI 379–496)–678 (95% CI 612–744) cases with the corresponding mortality rates varying between 15.1% (95% CI 13.7–16.6) in high-income to 34.2% (95% CI 29.5–38.9) in low-income regions [9,14]. These are age-adjusted weighted averages by country of residence.

Although age-standardized incidence declined by 37% and mortality by 52.8% between 1990 and 2017 [14], population growth and ageing have, to some extent, negated these improvements, as more people are affected.

#### 3.1.2. Sepsis in HICs vs. LMICs

The disparity in the burden of sepsis between high-income countries (HICs) and low–middle income countries (LMICs) is alarming. The heaviest load was borne by countries in sub-Saharan Africa, South Asia, Southeast Asia and East Asia [14]. This disproportionately high burden is in part because of a combination of high endemic infectious disease prevalence and poor access to healthcare, as well as deficiencies in under-resourced referral systems. HICs, for their part, have seen a reduction in case fatality rates thanks to better surveillance and diagnostics that have enabled patients to be treated earlier. Yet, in HICs, the hospital mortality for severe sepsis is still 15–25%, and higher for septic shock, with a 30–40% hospital death rate, but is also highly variable amongst different countries and individual centres [15]. When pooled, the case fatality rate was 18.7% (95% CI = 16.5–21.0) for HICs and 33.9% (95% CI = 30.1–37.8) for LMICs among income-level groups with a statistically significant discrepancy between them (*p* < 0.001 by chi-squared test of proportions).

#### 3.1.3. Vulnerable Populations: Neonates, Pediatrics, the Elderly, and Chronic Illnesses

Beyond the general burden, subpopulations are disproportionately affected. The overwhelming lion’s share of cases of severe sepsis globally is contributed by newborns and toddlers under five years of age, where more than 20 million cases are estimated to occur annually, 3 million among children aged below 5 years and another 5 million in children and adolescents aged between five to nineteen [9,15,16,17]. Pediatric sepsis incidence and mortality differ markedly from age-adjusted rates in older adults; among patients older than 65 years, mortality can reach 35% [15,17]. Thus, in such scenarios of low prevalence and absence of previous exposure to severe forms of the disease, the individuals suffering from chronic comorbidities such as malnutrition, HIV, diabetes, and tuberculosis (TB) are at higher risk of developing sepsis and experiencing more severe outcomes, particularly in low- and middle-income countries (LMICs), where these conditions are highly prevalent, and healthcare access is limited.

#### 3.1.4. Limitation in Data Collection and Reporting Bias

Although modelling approaches are increasingly sophisticated, empirical primary data remain sparse and are often of limited quality. The diagnostic accuracy for sepsis is variable in most countries’ administrative databases, and the prospective monitoring of sepsis is rare in non-tertiary centre settings [13]. Much of the incidence modelling has been led by a few countries in which rates are reported in comprehensive healthcare systems, leading to underestimates of LMIC estimates and over-representation of HIC patterns. It is also further complicated by retroactive reconciliation of different coding systems (ICD-based definitions of sepsis vs. Sepsis-3 criteria). Temporal changes, for instance, may depict changes in definition rather than true epidemiological movement [14]. Currently, there is little available data on outpatient sepsis, community-treated sepsis or sepsis in rural or remote populations.

### 3.2. Recognition Frameworks in Practice

Identifying sepsis promptly is the critical first step toward appropriate treatment, and, although sepsis screen tools seem to be a reasonable approach, sensitivity and specificity still present challenges. The qSOFA, developed with the Sepsis-3 consensus to identify sepsis quickly at the bedside in the non-ICU setting, has low to moderate specificity (83–91%) (11 and references within) but even lower sensitivity and consistently misses a substantial proportion of cases when used as the sole screening tool [18,19]. qSOFA had a sensitivity of approximately 50% in a pooled analysis for outpatients and ED (specificity approaching 80%), resulting in an AUROC of 0.74, and is insufficient for early screening of no-symptoms risk stratification patients [7,8]. Multicenter cohort data from published ED cohorts concur that qSOFA ≥ 2 discriminates better for risk of mortality than SIRS ≥ 2 (adjusted OR 4.6 versus 1.7) but is low in sensitivity (55%) and conversely with SIRS being higher in sensitivity (88%) but poor in specificity (19%) [12] (Table 1).

**Figure 2:**
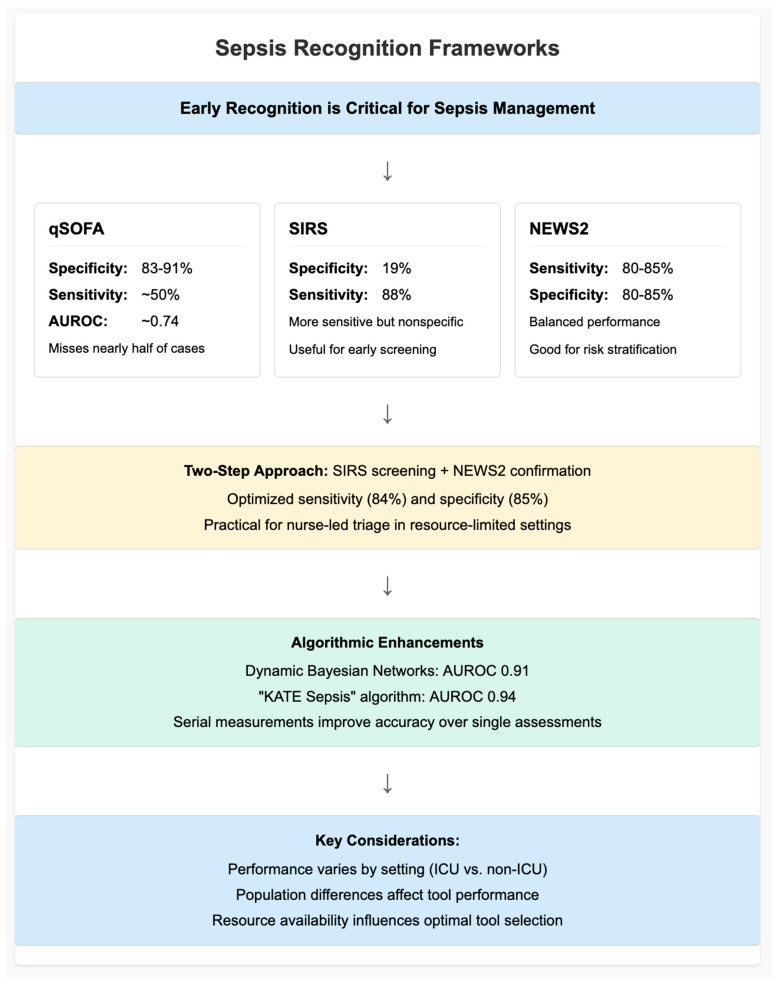
Conceptual overview of sepsis recognition frameworks. Quantitative diagnostic metrics (sensitivity, specificity, and AUROC) have been standardized and are reported. This diagram illustrates the clinical application pathways for qSOFA, SIRS, NEWS2, and machine learning models across pre-hospital, emergency department, and ICU settings. The colour gradients indicate sensitivity ranges based on our analysis of published validation studies [13,18,19,20]. Arrows indicate recommended patient flow pathways based on initial screening results.

NEWS2 by comparison provides a better trade-off in performance with sensitivity and specificity around 80–85% with a body mass of results that indicates it might work well as an initial instrument for risk stratification in general wards or community hospitals when the key objective is to identify “risk” as opposed to sensitivity [9,14]. More recently, a SIRS-based two-step triage strategy followed by NEWS2 confirmation was also successful in reanalyzing a resource-limited hospital’s dataset with improved sensitivity (84%) and specificity (85%), which may be feasible for bedside nurse-led triage when high-quality testing is not available [9] (Figure 2).

The diagnostic capability and clinical usefulness of sepsis biomarkers differ considerably across clinical environments. The best evidence of PCT is in emergency departments and ICU settings to help in early identification of bacterial infection and stewardship. Presepsin (sCD14-ST) is promising, as it increases rapidly in systemic inflammation and is useful, especially on early presentation to the ED. However, these biomarker results may be confounded in patients with renal impairment (Table 1) [8]. Neutrophil CD64 FcγRI receptor expression provides excellent diagnostic accuracy in ICU settings, including adult and pediatric ICUs, where prompt innate immune activation is especially useful. This context-dependent discrepancy emphasizes the need to distinguish biomarkers according to a clinical scenario rather than interchangeability. In contrast, hierarchical reference meta-analyses found that the diagnostic performance of qSOFA and SIRS was more variable between LMIC settings: the sensitivity (pooled from independent studies) of qSOFA was 72% (ICU) to 61% (non-ICU) and specificities were low (34%) even after exclusion of (referral or selective) bias; whereas SIRS had high sensitivity (88%), but low specificity [6,13]. This variation not only indicates differences in characteristics of tools, but it also indicates heterogeneity in study populations and times of measurement with respect to the baseline mortality risk, both of which are important when choosing or validating a screening score in a certain setting [13].

Beyond traditional clinician-led recognition approaches, algorithmic and machine learning models offer opportunities to improve early detection. In the ICU, a dynamic Bayesian network (DBN) model, based on physiological time-series data, has outperformed conventional scores in AUROC metrics, at a value of 0.91 compared to the SOFA (0.843), qSOFA (0.66), MEWS (0.73), and SAPS-II (0.77) at admission in the prediction of mortality [21]. In the context of the latter, machine learning models like the “KATE Sepsis” algorithm have achieved higher AUCs of 0.94 when used for triage in EDs, with a sensitivity of 71% and specificity of 95%, but without external validation across various global settings [22]. However, we should consider that these models are still largely experimental, and caution should be exercised due to limited external validation and risk of overfitting, particularly in LMIC settings where data may be sparse. The performance of the score is also influenced by the phase in which it is then applied. Derivation of serial, qSOFA, NEWS2, and SIRS scores while in the ED appears useful to further enhance the prognostic value due to increased sensitivity in recognition of deterioration, particularly with the NEWS2, where scores at maximum derangement improved prognostic fit once, and possibly better reflect insidious organ failure [23]. These findings suggest that repeated assessment and adaptive decision-making are essential, as score thresholds should not be considered static. In addition to statistical performance, the practical usability of these tools is important. The criteria for SIRS are difficult to apply and are insensitive, but they provide a laboratory-independent protocol for early antibiotic treatment. qSOFA can be rapidly performed at the bedside, which may, however, under-detect sepsis, particularly in children, elderly patients, or in LMIC due to heterogeneity of comorbidities and variation in physiological measures affecting its performance. NEWS2 and MEWS, while more nuanced, also rely on systematic recording of vital sign data with evidence-based cut-offs. NEWS2 has undergone several adaptations for UK use but is less well-validated in the international setting [14].

Meta-analyses comparing qSOFA, SIRS, and NEWS2 are limited by inconsistent inclusion criteria, variable baseline mortality risks, and timing of measurement, which confound pooled performance estimates.

### 3.3. Biomarkers and Rapid Diagnostics

Early identification and treatment of sepsis are based on early recognition and on diagnostic biomarkers, as well as quick pathogen detection, which will contribute to clinical scoring systems and aid in antibiotic therapy. The most studied biomarker is procalcitonin (PCT). Systematic reviews in the literature published until recently show that the PCT sensitivity for bacterial sepsis, as opposed to non-infectious inflammatory states or SIRS, is 77% and for specificity, is 79% [24], and that the performance of the PCT is influenced by the population studied, contraindications, and the clinical context. A pooled diagnostic meta-analysis (*n* = 23,452 patients) reported an AUROC of 0.86 (95% CI 0.82–0.90) for procalcitonin in distinguishing sepsis from non-infectious SIRS, while presepsin achieved a pooled AUROC of 0.84 (95% CI 0.79–0.88) [24,25]. These values confirm the complementary diagnostic role of biomarkers when integrated with clinical scoring systems (Table 2).

A more recent meta-analysis ended up with a sensitivity of 70% and specificity up to 87%, which is once again indicative of its usefulness only as an add-on diagnostic marker and not as the sole diagnostic [26].

Recent studies suggest that combining biomarkers—such as PCT with presepsin or CD64 with lactate—may improve early diagnostic accuracy, although most combinations still require external validation in diverse populations. Evidence from 2022 to 2024 meta-analyses highlight the potential of integrated biomarker panels in complex presentations such as ED triage and immunocompromised patients. Despite their promise, clinical uptake remains heterogeneous, particularly in resource-limited settings, where cost, assay availability, and turnaround times pose substantial barriers. These gaps emphasize the need for implementation research to determine how biomarkers can be optimally integrated into context-specific sepsis pathways.

**Figure 3:**
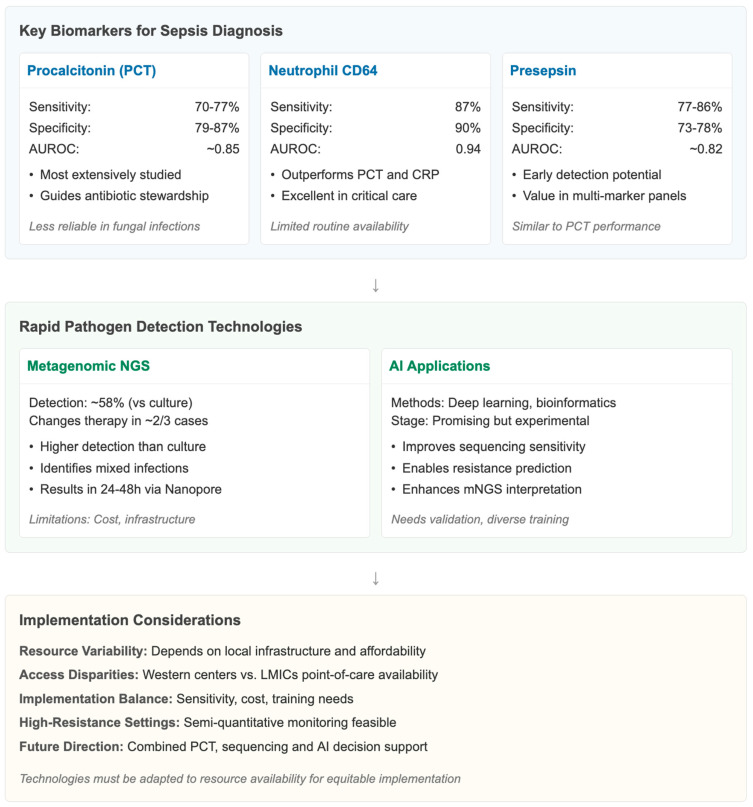
Diagnostic performance of key sepsis biomarkers. This original forest plot compares the pooled area under receiver operating characteristic curves (AUROC) with 95% confidence intervals for procalcitonin, presepsin, neutrophil CD64, and CRP. Data synthesized from our analysis of meta-analyses by Wacker et al. [24], Zhang et al. [25], and Wang et al. [27]. The visualization demonstrates the superior diagnostic accuracy of neutrophil CD64 compared to other biomarkers.

Finally, neutrophil CD64 excels PCT and CRP even in critically ill patients, with a pooled sensitivity of 87%, pooled specificity of 90%, and AUROC of 0.94, which was supported by a subgroup analysis of this biomarker in multiple meta-analyses [27] (Figure 3).

Biomarkers are widely accepted since they provide good diagnostic accuracy; however, cut-offs vary, and the sensitivity of testing is far from ideal. It increases markedly within 3–6 h after exposure to bacteria and decreases in a well-described manner in correlation with the clinical course of the patient, thus being a helpful tool in antibiotic stewardship algorithms, but could be less useful in localized fungal as well as in localized parasitic and non-infectious inflammatory disease, where it could be elevated [28]. Another potential biomarker is presepsin, which has pooled sensitivity ranging from 77% to 86%, specificity between 73% and 78%, and performance close to that of PCT. Hence, the performance of PCT can serve as a multilevel panel for the early detection of sepsis [25].

In recent years, the progress in rapid detection of pathogens has been groundbreaking, of which metagenomic next-generation sequencing (mNGS) is the most unsettled. Multiple studies, which cover single-centre cohorts to multicenter trials, have demonstrated that mNGS achieved a detection rate superior to the conventional culture in identifying pathogens and an ability to detect mixed infections, fastidious, or viral agents that are beyond the scope of standard diagnostics [11,29]. A retrospective cohort study found blood mNGS to be positive 58% of the time and guided antimicrobial intervention in 2/3 patients, but it was of high utility [30]. In prospective clinical trials, mNGS reduced the time to pathogen identification in the Nanopore mNGS cell-free DNA workflow by at least one day following sample collection (24–48 h) [31,32] and better pathogen detection than culture identification in respiratory samples [33]. Nevertheless, there are concerns about the cost, bioinformatics resources and false positives, or the presence of contaminants in samples. At a more advanced stage, rapid mNGS is supported by AI-assisted interpretation. Large-scale testing of deep learning models linked with sequenced and clinical data is scalable for pathogen detection and resistance prediction, yet it is still experimental. While models such as MG2Vec hold promise for unsupervised pathogen identification directly from raw sequencing, they have seen limited real-world validation in diverse settings [32]. At a health systems level, there may be variable utility for biomarker and mNGS strategies depending on local infrastructure and costs. PCT-guided algorithms to decrease antibiotic duration at Western centres [11,28] may need to be considered according to the type of assay employed, but effects are unlikely to be transferable to as great an extent, given the absence of point-of-care PCT assays and mNGS platforms in resource-limited countries. Biomarker-directed stewardship with semi-quantitative PCT or lactate could still be used in high-resistance area settings, or in the case of limited lab and not used in association with a clinical protocol.

In a clinical scenario, sensitivity, specificity and even some case availability, cost for implementation and training may be required for these technologies to be implemented. PCT is useful, but should be interpreted in the clinical context, which might be better served by panels including presepsin. mNGS offers better pathogen coverage, although cost, turnaround time, and informatics infrastructure for data interpretation are challenges. The combination of PCT monitoring, rapid sequencing and AI-supported decision-making is future-oriented and will have to be adapted to the available resources and validated in various global settings. Many biomarker studies are single-centre, use non-standardized assays, and report optimistic diagnostic performance that has not been reproducible across diverse settings.

These diagnostic advances align with Proposition 2, which positions infrastructure readiness—and not technological capability alone—as the constraint governing their real-world impact.

### 3.4. Time to Optimal First Management

Time to treatment is a key determinant of outcome in sepsis care, yet turning that imperative into practice globally has been challenging. Observational composing benchmark trials have consistently demonstrated the truth that every hour of antibiotic delay increments septic shock-related mortality by 6–10% [33,34]. While the Surviving Sepsis Campaign (SSC) has issued an “Hour-1 Bundle” including antibiotic administration, blood cultures, lactate measurement, and at least 30 mL/kg crystalloid fluid resuscitation in the first hour after recognition of sepsis [35], adherence rates are very variable and most studies report that fewer than 50% of patients receive guideline-concordant therapy, even in high-income hospitals.

This is consistent with fluid resuscitation algorithms that target 30 mL/kg of crystalloid fluids over 3 h as part of hemodynamic stabilization along with adjuncts such as lactate clearance or dynamic parameters [35]. But the opposing issue may still risk harm: uncontrolled microvascular fluid loss and positive fluid balance have been argued to correspond with organ edema in studies, which reported detrimental effects regarding too-aggressive fluid resuscitation [36]. Titrating could also be difficult since we had no “baselines” (as there were only follow-up measurements for a handful of patients) and even small changes in lactate over time could not be null or “reasonable” as even a small variation in lactate could suggest that a patient was at risk of a dramatic lactic acidosis. In diseases like abdominal or urologic, or soft-tissue sepsis, source control must be achieved promptly (ideally within the first 12 h), as delays are related to increased mortality and longer LOS [37]. Nevertheless, delays exist in practice due to diagnostic uncertainty, such as follow-up or repatriation to the LMIC rural areas where surgical services are centralized and limited [38].

Studies involving different multicentric facilities showed that compared to the practice using manual recognition, the hospital with electronic health records and a sepsis alert system has shorter “door-to-antibiotic” times (~50 min) and a lesser effect in terms of the progression into septic shock. But in practice, false alarms and alert fatigue cause these systems to become largely useless [39].

Uniform CPGs and bedside checklists have been pilot tested in QI work. In the UK, the introduction of the “Sepsis Six” bundle in emergency departments was associated with a fall in hospital mortality from 29% to 17% over 12 months and reduced ICU length of stay [40]. There are some established programmes in middle-income countries with some improvements in process of care indicators as time to first antibiotic delivery, % volume and type of fluid delivered in goal, but have been resource dependent and productivity has often been monitored in relation to staff activity and availability [41].

While we know the rapid antibiotics, early source control, and fluid targets are all good based on evidence, there is a price to pay. Indeed, historical data indicate that strict use of time metrics without the ability to use clinical judgement is linked to over-treatment with broad-spectrum antibiotics, boluses for fluid resuscitation in patients at risk for volume overload, or antibiotics with potential side effects in cases of non-septic SIRS [42]. As such, stewardship teams must weigh the toughness with the diagnostic accuracy to minimize “collateral damage” related to antimicrobial resistance or procedural harm.

Baseline infrastructure—often at a stretch already at baseline in LMICs—may further delay services. Overcrowding in the triage system, its excessive workload, lab bottlenecks and erratic availability of drugs in sub-Saharan Africa increase the median time to administer antibiotics from 3 to 4 h [43]. In addition, transport delay, uncertainty of diagnosis and the need to wait for the referral, particularly in rural areas or when referrals are initiated from specialized primary care mode, which is hard to monitor.

At the global level, combined approaches of applying clinical guidelines and introducing system-wide interventions are required. These implementation strategies include electronic medical record-based recognition and alert systems, bundled checklists with continued education and audit–feedback, rapid laboratory support or bedside lactate devices, and stewardship oversight to minimize overuse. Successful programmes strike this balance through a sense of urgency, urging empiric antibiotic therapy and flexibility, where prompt dose escalation to an indications-based approach once diagnostic test results have resolved concerns about the anticipated etiology of infection and providing fluid protocols for individuals according to their risk status or comorbidities. Although SSC bundles are widely recommended, several components—particularly fluid targets and timing of interventions—are derived from observational data with significant confounding.

Observed delays in treatment reflect systemic process inefficiencies, supporting Proposition 3.

### 3.5. Antimicrobial Therapy and Stewardship

Empirical antimicrobial therapy is still the most timely and pivotal intervention in the management of sepsis; however, urgency needs to be balanced against the risks of increasing AMR from patients to the community or even promoting AMR locally. Two high-quality observational studies have shown that waiting for the serum antibiotic level does not seem to delay appropriate therapy much, and in the one study that compared mortality between those who did and did not, the delay had less impact on mortality than incorrect first antibiotic choice, which is associated with not covering a likely pathogen [44]. But we are not making things better by using unnecessarily broad-spectrum antibiotics or by continuing with empiric therapy longer than necessary; we just drive-up resistance post-treatment. Sepsis “pathways” with stewardship elements are increasingly considered essential. Procalcitonin-driven antibiotic algorithms in the context of tertiary care facilities have resulted in early discontinuation protocols and reduced duration of antibiotics (by 1–2 days) with minimal effects on mortality or readmission rates [45]. While third-generation cephalosporins are infrequently used as empirical therapy in neonatal units worldwide, in LMICs, up to 45% of Gram-negative pathogens are resistant to aminoglycosides and third-generation cephalosporins [46], further emphasizing the need to compare custom-tailored antibiotic regimens with local rather than international antibiograms as opposed to international recommendations. The emerging concept of site-specific stewardship in the post-NGSP era has provided a powerful impetus for two-stage antibiotics, or rather initial broad coverage followed by rapid de-escalation armed with meaningful culture or biomarker data. A multicenter prospective cohort in high-income settings found that integrating point-of-care testing with stewardship oversight led to antibiotic initiation within 2 days being de-escalated in 65% of sepsis cases without affecting organ dysfunction metrics or duration of stay in the ICU compared with standard care [47].

Implementation remains uneven. A quality improvement intervention in Brazilian public hospitals showed that including stewardship protocols within sepsis bundles was associated with increased adherence to guidelines, a 20% reduction in use of broad-spectrum carbapenems, with a modest impact on mortality benefit (from 32 to 28%) [48]. These initiatives may encounter several barriers, such as the absence of stewardship infrastructure, lack of expertise in training and inadequate microbiology support, in addition to other challenges such as in LLMIC and could be faced. There have also been changes in local empirical therapeutic paradigms due to the emergence of carbapenem-resistant Enterobacteriaceae and ESBL organisms [49], with evidence showing empirical rates of >10% in many batches.

Instructors from across the world have reacted to develop guides. The 2021 update of the Surviving Sepsis Campaign, however, emphasizes that antibiotic selection should be patient-centred and should reflect institutional patterns of epidemiology and susceptibility and suggests reassessing them within the first 48–72 h on clinical grounds or isolating the pathogen [36]. Using simple syndromic approaches to common causes of URTI and low-cost point-of-care tests, such as rapid immunoassays, offers only modest reductions in antibiotic consumption. Guidance in the WHO manual reinforces the very same tenets that we continue to suggest to this day: short empirical duration and pathogen-directed switch therapy when microbiological or clinical clarity is achieved to better facilitate de-escalation in low-resource settings [50].

The connection between stewardship and health system preparedness is paramount in the end [51,52,53]. E-prescribing plus antibiogram-driven decision support can decrease empirical inappropriateness and promote early de-escalation in settings based upon a strong background IT system, flawless lab interfacing and enslaved prescribers. Paper-based systems are the norm for MWU guidelines in many LMIC hospitals, and there will be no access to microbiology feedback to allow timed de-escalation to be applicable.

The dependence of stewardship success on institutional systems, audit loops, and laboratory capacity illustrates Proposition 4.

### 3.6. Organ Support and Adjunctive Care

In the setting of early sepsis, trajectories of patient management are frequently shaped by the emergence of organ dysfunction, and the possibility of organ support and adjunctive strategies is thus a crucial aspect of contemporary sepsis management. Hemodynamic support may require administration of norepinephrine for initial vasopressor support in the presence of catecholamine-refractory arterial hypotension, which, when combined with volume loading, has resulted in the return of hemodynamic status (MAP ≥ 65 mm Hg) on large, randomized, controlled trials with better survival outcomes and fewer arrhythmogenic events versus dopamine [53]. In patients not reaching target MAP, adjunctive vasopressin is a consideration, but VASST data indicate that its effect is limited, and it is not associated with overall improvement in outcomes [54].

The need for mechanical ventilation in sepsis-induced acute respiratory distress syndrome (ARDS) demands lung-protective strategies: tidal volumes of ≤6 mL/kg predicted body weight, plateau pressures below 30 cm H_2_O and optimal PEEP levels that effectively avoid ventilator-induced direct pulmonary injury and risk of death [55].

The requirement of mechanical ventilation in sepsis-induced ARDS requires lung-protective ventilation with tidal volumes of ≤6 mL/kg predicted body weight, with a plateau pressure PaO_2_/FiO_2_ ratio < 150. This would render an affordable, non-invasive approach feasible and testable even in resource-limited environments [56].

AKI frequently occurs after Sepsis (S-AKI), and renal replacement therapy (RRT) is frequently initiated, yet the timing of RRT commencement is uncertain. The most recent and randomized trials (STARRT-AKI) have demonstrated no survival advantage with early initiation and a higher degree of RRT-dependency among survivors; it is therefore reasonable to preferentially use a more conservative “watchful waiting” strategy approach in most situations [57]. Furthermore, there is evidence that continuous renal replacement therapy (CRRT) may be preferred in hemodynamically unstable patients, although the choice between CRRT and intermittent dialysis therapies remains challenging, especially in low- and middle-income countries (LMICs).

Contradictory results are observed regarding adjuvant therapies, such as corticosteroids, vitamin C, and hydrocortisone in combination therapy. Whilst interest in the use of hydrocortisone in septic shock has waned a little more of late, largely in response to the rather modest mortality benefit seen on trials such as ADRENAL and APROCCHSS, there are certainly very divergent views on this treatment at a global level, as evidenced from multiple meta-analyses [58,59,60,61,62]. The VITAMINS study, which was a multinational trial, demonstrated that the use of vitamin C/hydrocortisone/thiamine therapy with usual care does not result in better resolution of shock or survival [63]. Recombinant activated protein C (drotrecogin alfa) was also withdrawn after landmark trials were not able to show the extent of benefit in broader patient groups. Prone position and conservative fluid management can mitigate VILI, and delayed RRT can eliminate complications related to the procedure itself. However, add-on therapies to include immunomodulation still require scrutiny, balancing anecdotal positivity with evidence from authentic, RCT-derived data.

Generalization to other domains is one of the fundamental problems. On the part of high-income countries, such organ support interventions (frequent SOC services with considerable worldwide availability) are offered according to infrastructure; assisted by multidisciplinary teams that are good at decision-making, they structure supply together with the demanded monitoring. What we are facing in HIC, which we typically do not see in LMIC, is a lack of ICU beds, functioning ventilators, and a structure for dialysis. At low-resource centres, these may have lower-cost alternatives, such as simplified ventilation protocols, early mobilization, or fluid balance monitoring, that can bring the facility close to a best-practices level. There is inconsistent application and evidence of adjunctive interventions, including low molecular weight heparin or plasma exchange, in sepsis-induced coagulopathy or DIC with additional risks in resource-scarce settings [60]. This is less enforceable than prevention (we see it to a lesser extent even for diarrhea control) but is none the less important for other outcomes and includes nutritional supplementation (including also feed delivery method) and glucose control, though glucose control is often irrational; hyperglycaemia magnifies mortality risk when poorly controlled but tight glucose targets may result in increased episodes of hypoglycaemia in settings without continuous monitoring [61].

### 3.7. Clinical Guidelines and Their Uptake

Despite guidelines providing recommendations for sepsis care, practice patterns vary considerably across regions and healthcare settings. Bundle interventions in the most recent Surviving Sepsis Campaign (SSC) guidelines, first published in 2004 and last updated in 2021, are early administration of antibiotics with either hemodynamic support or source control or organ support titrated according to a point scheme calibrated on trial evidence [62]. While the use of these guidelines in a routine setting has proven to be beneficial in some clinical scenarios, the real-world application is still incomplete. The heterogeneity in guideline adoption has been attributed to the complexity of the recommendations, non-universal adoption by hospitals, perhaps due to institutional inertia, resource constraints and competing clinical priorities [63]. In contrast, the UK National Institute for Health and Care Excellence (NICE) guidelines recommend risk stratification with the National Early Warning Score (NEWS2), early antibiotic initiation in high-risk patients, and delayed/standby prescription or no antibiotic treatment versus immediate antibiotic therapy in low-risk patients to reduce inappropriate antibiotic exposure [64]. This indicates a more conservative antibiotic policy than the SSC “Hour-1 Bundle” and a key point of divergence in the approach to practice: SSC advocates rapid broad administration and NICE individual clinical assessment with faster or slower provision according to severity of illness.

The World Health Organization (WHO) has tiered guidelines for low-resource settings, emphasizing can-be-recognized and treated at scale interventions; these include early fluids, antibiotics, and biomarker-guided monitoring that do not require advanced lab and ICU care [65]. This WHO guidance is realistic, bridging implementability and efficacy, promoting de-escalation when diagnosis is possible, and integrating sepsis response within maternal, neonatal, and antibiotic stewardship platforms [65].

A comparison of SSC, NICE, and WHO demonstrates significant congruence around some core principles (early recognition, prompt antibiotics, fluid resuscitation) but substantial divergence in the specifics of implementation and operational freedom. An international survey across six continents found SSC implementation to have penetrated ~60% of third-level centres in high-income countries, and 15–20% of hospital facilities in LMICs have implemented tools that are either simplified versions or derivatives of WHO guidance [66]. The complexity of SSC bundles in real practice (multiple timed goals and lab-based dosing thresholds) may represent a large hurdle to implementation in settings without electronic order sets, real-time lab trace or sepsis champions.

On a policy level, there is also a significant impact on the uptake of guidelines. In countries that have incorporated sepsis as a dedicated component of national health plans, sepsis protocol implementation at the hospital level will need to happen faster and may be somewhat performance-based at least for funding [67]. Whereas in countries where the burden of sepsis remains underestimated, guideline implementation appears to be non-mandatory and unsupervised, resulting in poor implementation activities and minimal process change.

Equity challenges also arise in guideline implementation. The SSC and NICE bundles are largely designed for high-resource environments and assume prompt access to ICU-level treatment and care, electronic monitoring, and advanced imaging, while WHO materials are intended to counter those biases by providing tiered guidance that can be applied to primary facilities. Therefore, alterations without abandonment of central principles of therapy represent a tremendous challenge.

### 3.8. Examples of Successful Sepsis Quality Improvement Initiatives

Several large-scale sepsis quality improvement (QI) initiatives have demonstrated measurable benefits in early recognition, timeliness of treatment, and survival outcomes. The UK Sepsis Six programme, implemented nationally through the UK Sepsis Trust, significantly reduced mortality and length of hospital stay by promoting rapid bedside delivery of oxygen, fluid resuscitation, and antibiotics within one hour of suspicion [68]. Similarly, the Surviving Sepsis Campaign (SSC) bundles—adopted in over 70 countries—have led to substantial improvements in compliance with evidence-based sepsis management, with reported relative reductions in mortality ranging from 12% to 25% following structured implementation and audit–feedback mechanisms [35]. In the United States, statewide programmes such as the New York State “Rory’s Regulations” mandated hospital-based sepsis protocols and public reporting, resulting in earlier antibiotic administration and decreased sepsis-related deaths [69]. In Australia and New Zealand, the ANZICS Sepsis Breakthrough Collaborative has emphasized multidisciplinary engagement and data-driven improvement, demonstrating sustained increases in compliance with 1 h treatment targets. These examples illustrate how coordinated, system-level initiatives—integrating training, real-time monitoring, and accountability—can translate clinical guidelines into measurable outcome gains. Such models provide transferable lessons for low- and middle-income countries (LMICs) aiming to strengthen sepsis recognition and management capacity. Education and training is another significant factor. Targeting implementation of a guideline to organizations ensured that pre-implementation factors, such as implementation by local educators (as would be expected) and simulation-based training combined with continuous knowledge of performance, contributed to higher adherence [70] to the guideline and more favourable clinical outcomes, such as time to antibiotic delivery and bundle completion. Facilities where models of dissemination are passive, such as emailing guidelines or expecting clinicians to adopt new knowledge themselves, typically have low uptake and modest clinical impact.

### 3.9. Digital Tools and AI-Enabled Recognition

There is growing evidence on digital augmentation of clinical diagnosis in sepsis by means of non-invasive imaging and use of machine learning models, which holds promise in future care of septic patients. These are measured against evidence from advanced analytics that are superior to traditional scoring systems when properly validated and integrated into healthcare system operations. A DBN model, using structured physiological and clinical data, and training on the same cohort, had excellent prediction performance (AUROC, 0.91) for predicting mortality among patients admitted to the ICU with suspected infection, with results being better than SOFA 0.843, qSOFA-0.66, MEWS-0.73 or SAPS-II-0.77 [24]. Other models, such as InSight, also operated effectively even with very little vital sign data (AUROC ≈ 0.78) and included missingness as high as 60% Airi Ubaid validated against prospective clinical implementation [26].

A network meta-analysis of more than 450,000 sepsis cases and 256 predictive models recently demonstrated that machine learning algorithms—and especially the neural networks and decision tree ensembles—that they studied outperformed the traditional scoring tools in all scenarios (pooled AUROC ranging approximately from 0.75 to 0.96) [13]. This can be the basis for early prophylaxis and fast-track care interventions, as some of these models can predict sepsis hours earlier than traditional tools.

Non-invasive imaging biomarker methods have undeniable operational advantages. This will make a cheap, non-invasive test viable and testable even in low-resource settings.

Multiple HSI-based studies for developing non-invasive microcirculatory pattern recognition have demonstrated that there are unique spectral characteristics ([Hb]) and reduced SpO2 associated with sepsis in both fingers and palms [3], permitting the non-invasive, early, and objective mapping of changes in response to sepsis physiology. These imaging biomarkers have the ability of early stratification and may allow non-invasive monitoring.

But achieving such a result at scale with an AI tool is incredibly difficult. However, few of these models are externally validated in a variety of healthcare settings and may not perform well when applied to other hospitals with different patient populations, EHR infrastructures and clinical practice patterns [4,13]. Alert fatigue is another issue: the clinical value of algorithms that perform well (AUROC—0.80 and higher) can be limited by false positives when they erode trust or add excessive clutter to thought-critical workflow [9]. So, bringing in the same alerts into EHR must be sensibly performed; it must be configurable, and the personnel who use them should be trained before they can use.

There is concern regarding biases overfitting in terms of preferred patient populations, opacity of “black box” models, and dependence on proprietary tools that may not scale evenly. In addition, technology readiness varies. Although the HSI platforms are resource-intensive compared to ICU monitoring, they do have hardware costs and staff training associated with them. Where scales are found to be used at scale in wards, general hospitals or health centre outposts, they must undergo adaptation of their validation and deployment study (contextualization) [5].

The results of this review have important implications for clinical practice and health policy. Enhancing national sepsis control programmes should be based on standardized surveillance platforms developed and scaled up in line with the recently launched WHO Global Sepsis Report, and supplemented by benchmarks such as sepsis metrics embedded in standard hospital performance indicators. Clinically, integrating sepsis recognition algorithms, point-of-care diagnostics, and antibiotic stewardship bundles into emergency and primary care workflows is expected to enhance early identification/surveillance, leading to better outcomes. For policymakers, allocating resources to train the workforce, ensure prompt access to diagnostic testing, and develop infection prevention infrastructure is critical (particularly in LMICs), which may help address global disparities in sepsis care. Lastly, such observations may help health ministries and professional societies to construct flexible, context-specific recommendations for sepsis care that will narrow the chasm existing between evidence and practice.

The future research priority is to develop high-quality multicentric data to inform a more global approach to sepsis surveillance and provide better representation of community-acquired and non-hospital cases, which are currently undersampled by existing models. Biomarker panels, metagenomic sequencing, and AI-based transit options need to be prospectively validated across resource settings to determine reproducibility and scalability. Additional research to assess the cost-effectiveness and actual application of stewardship bundles and digital alert systems in HICs and LMICs is also warranted. Third, multidisciplinary health systems research—epidemiology, informatics, and the policy sciences—must investigate context-specific models of preventing sepsis, early detection (and not too late to intervene), and equitable management at a population level.

Our structured review adds novel perspectives to an updated and comprehensive overview of multidisciplinary approaches to sepsis recognition and global implementation. Unlike previous systematic or consensus reports, our work identifies relatively unexplored intersections between (1) epidemiologic data; (2) diagnostic performance; (3) digital innovation; and (4) the translation of policy needs, positioning sepsis as a systems-level problem that demands a bundled (diagnostic-policy) coordinated response.

The upcoming concept of patient subphenotyping based on molecular signatures and biomarkers holds great potential in the context of more individualized sepsis therapy. Sepsis trials are now moving away from the broad syndromic definitions towards a “theragnostic” paradigm, where interventions are directed to certain patient populations who have been characterized for treatable traits [71]. This approach could potentially address the long-time sepsis population heterogeneity issue that has confounded numerous clinical trials. Such precision methods could enhance digital tools and AI-powered characterizations of who has sepsis by determining not only that someone has it, but also which particular interventions might help a patient based on his or her distinct biological profile.

### 3.10. Post-Sepsis Syndrome and Long-Term Outcomes

Severe sepsis survivors frequently suffer enduring sequelae denoted as post-sepsis syndrome encompassing a range of debilities, both physical and mental, that may persist for months or years after hospitalization. Physical consequences are chronic fatigue, muscle weakness, neuropathy, and reduced mobility resulting in long-term disability with a significant deterioration in quality of life. Cognitive sequelae, including deficits in memory and executive function, manifest in 30–40% of survivors and are linked with persistent inflammation, critical illness-related brain dysfunction, and hypoxic insult [72]. Psychological sequelae such as PTSD, depression, and anxiety are also frequent and may contribute to the worsening of functional decline [73]. Long-term mortality is still elevated and as many as 25–30% of sepsis survivors have died within a year of their index episode, generally from recurrent infections or cardiovascular disease [74]. Moreover, repeated admissions represent a marked economic and social impact, especially in low–middle-income countries (LMICs) with limited post-discharge follow-up options. These results underscore the need for initiating sepsis survivorship programmes, multidisciplinary rehabilitation pathways, and post-discharge surveillance as included components of national action plans on sepsis. Incorporation of prospective long-term outcome surveillance into sepsis registries may enhance our appreciation of the chronic sequelae and enable focused interventions.

### 3.11. Critical Global Domains: Prevention, Health Systems, Workforce, and Maternal/Neonatal Sepsis

From recognition and diagnostics, to therapeutics, digital augmentation and long-term care, our synthesis demonstrates that the key determinants of global sepsis outcomes are at a systemic level rather than the “bedside” alone. While previous reviews have summarized evidence within each silo, few explain how these components interact to create a systems architecture that drives survival. By codifying these in five explicit systems propositions, this review brings clarity to the reasons why clinical progress has been variable in its dissemination throughout the world.

Control of sepsis needs to be all-encompassing, beyond the immediate clinical management. Prevention interventions addressing pneumonia (e.g., pneumococcal, influenza, and RSV vaccination/immunization programmes), strengthening infection prevention and control (IPC) practices, improved water/sanitation infrastructure, as well as early community management of common childhood infections, should continue to form the cornerstone of decreasing global burden. Despite obvious advantages, use in practice varies widely, and adoption is poor; this may be particularly true for low-resource settings where IPC compliance, antimicrobial resistance and availability of vaccines are continuing issues. Beyond the acute care episode, there is growing recognition of the crucial role of post-sepsis care. Survivors often have persisting morbidity, with fatigue, neuromuscular polyneuropathy (weakness), cognitive impairment, anxiety and depression, and recurrent infections limiting their quality of life; these morbidities enhance long-term mortality risk. These sequelae are particularly amplified in LMICs where access to rehabilitation services, follow-up clinics and mental health support are limited. Formal survivorship programmes and post-ICU clinics are increasingly recognized as important elements of sepsis care. Health security systems and infrastructure directly impact sepsis outcomes, with many LMICs restricted by scarce budgets, lack of ICU beds, uneven laboratory capacity, and oxygen supply limitations limiting access to life-saving antimicrobials. High cost-sharing usually postpones care-seeking and causes excessive mortality. Sustainable investment in emergency care, diagnostic laboratories, referral systems, and critical care capacity are required to close global inequalities. Likewise, significant workforce deficiencies mitigate the adoption of sepsis guidelines. Inadequate nurse-to-patient ratios over safe levels and a lack of training in triage, early recognition, and emergency care lead to late identification. Effective models, including WHO’s Emergency Triage Assessment and Treatment (ETAT), midwife-implemented maternal sepsis bundles, simulation training for sepsis management, and audit–feedback cycles have demonstrated enhancements in capacity and utilization of evidence-based practices. Lastly, maternal and neonatal sepsis continue to be overrepresented in global mortality. Maternal sepsis contributes to about 10% of the global maternal mortality burden, and is frequently associated with poor obstetric care practices, postpartum infections, and inadequate IPC at delivery. Neonatal sepsis, accounting for 3 million estimated annual cases worldwide, is often associated with multidrug-resistant (MDR) Gram-negative organisms and still results in high mortality rates in several areas. Better understanding is needed for the impact of WHO-recommended pathways (clean birthing, intrapartum antibiotic prophylaxis, rapid recognition algorithms, early breastfeeding, and expedited referral systems) that, in seemingly high-quality studies, have shown some effectiveness but wide variation in implementation. Taken together, these domains highlight that sepsis is not only a clinical entity but also a systems issue. Prevention, survivorship, health system limitations, and workforce preparation for at-risk populations, particularly mothers and newborns, are critical for attaining equitable global progress of sepsis outcomes.

Collectively, these domains demonstrate Proposition 5: health system structure sets the upper limit for achievable sepsis outcomes.

### 3.12. Limitations

This review has several methodological and interpretation limitations. First, as a narrative review of literature, the analysis is inherently prone to selection bias and study heterogeneity. The latter includes variations in (1) diagnostic definitions (Sepsis-3 definition vs. ICD coding), which have a considerable effect on incidence estimates; (2) case ascertainment methods across studies and differences in patient populations under scrutiny. In addition, we may have missed some evidence from low- and middle-income settings due to the focus on predominantly English-language literature and because databases included were overrepresented by high-income countries. Third, simulated data or retrospective cohorts may include reporting bias, and the comparison between different studies should be performed with caution. Lastly, the applicability of our findings is limited by the diversity of healthcare delivery systems, diagnostic capability, and sepsis surveillance throughout the world. Despite these limitations, our overview gives a comprehensive and balanced summary of the current global status of sepsis in terms of epidemiology, recognition, and management.

As recently noted, the era of broad-spectrum “sepsis drugs” is now giving way to more selective, personalized interventions tailored to individual biological profiles, offering a more promising pathway for future therapeutic development, even in the absence of infection.

Another limitation is that, given the high level of heterogeneity in terms of study design (epidemiology, diagnostic performance/biomarker research, and implementation studies) for included primary studies, we did not use a uniform risk-of-bias or study quality tool. SANRA influenced the review design and reporting, but it does not assess the methodological quality of studies. The robustness of generated conclusions using different study designs with a wide range of metastases remains dubious.

## 4. A Systems Framework for a Sepsis Programme: Five Core Propositions

Integration of evidence in epidemiology, diagnostics, stewardship, organ support, digital innovation, and health system determinants indicates that sepsis response is influenced most from the system level as opposed to clinical advances only. From this review, we generate a systems framework with five components that organizes the structural drivers of global sepsis inequities.

**Proposition** **1.** *Recognition through systems, not tools in isolation, is associated with early identification.*

Model (qSOFA, NEWS2, SIRS) and biomarker performance vary by population, but system design—workforce density, triage pathways and confidence in documentation—determines when and how these tools are used.

**Proposition** **2.** *The diagnostic worth depends on infrastructure preparedness.*

High-throughput diagnostics (mNGS, AI-based models, biomarker multiplex panels) have a large role to play, but only if integrated into systems that can manage the procurement, laboratory turnaround time, interpretive expertise, and data governance they rely upon.

**Proposition** **3.** *Efficient process, not protocol, drives timely care.*

Delays in antibiotics, source control, and fluid delivery continuously reveal process bottlenecks (crowds, time to lab result, out-of-stock drug items, referral delays) and not a knowledge base gap.

**Proposition** **4.** *The effectiveness of stewardship hinges on institutional feedback loops.*

ASPs work where there are working audit–feedback cycles, antibiogram production, and e-scripting in hospitals: when absent in such system-wide nets, stewardship cannot fight AMR pressures.

**Proposition** **5.** *Maximum sepsis survival is limited by health system capacity.*

All the factors that statistically predict outcome are as follows: ICU access, oxygen availability, and their impacts on maternal–neonatal care quality. The way in which referral pathways are intertwined is more important than any one clinical intervention.

## 5. Key Conceptual Points from the Review of the Literature

The collective lessons from epidemiology, diagnostic performance, antimicrobial stewardship, and systems-level determinants provide four principal thematic messages which underpin sepsis outcomes worldwide:

- Diagnostic aids are required, although not repeated in early recognition.

Their real-world impact is determined more by access to trained staff and consistent capture of vital signs than raw test performance, laboratory, or triage. Not even advanced ones (e.g., NEWS2, PCT, CD64) can make up for weak system infrastructure.

- The success of antimicrobial stewardship is, at its core, a systems achievement.

There is a wealth of international evidence that stewardship interventions are most effective where there are hospital audit-and-feedback loops, microbiology support, electronic prescribing systems, and sufficient workforce capacity. Stewardship impact is severely limited by stockouts, diagnostic delays, and lack of monitoring in resource-poor settings.

- Health systems capacity is the limiting factor of possible clinical outcomes.

Regional variation in mortality is less explained by the variance of pathogens and more by structural determinants: ICU capacity, oxygen supply, referral systems, IPC implementation and maternal–neonatal care platforms. Ultimately, these things determine whether evidence-based practices will be implemented.

- New technologies (AI/ML, digital triage, automated alerts) expand rather than close system gaps.

AI solutions perform well under ideal conditions, but their success is contingent on routine data quality, interoperability, and expertise in the workforce as well as evaluation frameworks. Without such system support, these technologies risk exacerbating rather than resolving existing inequities.

## 6. Conclusions

Our review shows that sepsis is essentially a systemic condition with downstream consequences representing system-based causes: workforce numbers, diagnostic capability, surveillance infrastructure, process efficiency (or lack of), and stewardship integration. The five theses proposed in this manuscript present a new and unifying paradigm for addressing remaining inequalities and may inform future global strategies beyond current WHO and SSC recommendations. There have been great advances in diagnostics, clinical guidelines and digital tools, but their impact is spotty when applied in real-world situations because of a lack of access, training, and fidelity in implementation. This will necessitate the harmonization of evidence-based practices with health system realities such as flexible protocols, stewardship integration, and digital innovation. A concerted effort worldwide is required to achieve the WHO targets by 2030, reduce mortality, and manage sepsis equitably.

## Figures and Tables

**Table 1:** Summary of performance characteristics of sepsis recognition tools and AI-based models.

Biomarker	Primary Function	Sensitivity (%)	Specificity (%)	AUROC	Most Useful Settings
Procalcitonin (PCT)	Marker of systemic bacterial infection	70–77	79–87	0.82–0.86	ED; ICU; antibiotic stewardship
Presepsin (sCD14-ST)	Monocyte activation marker	77–86	73–78	0.79–0.88	ED triage; acute medical wards
Neutrophil CD64	Neutrophil activation receptor	~87	~90	~0.94	Adult and pediatric ICU
C-reactive protein (CRP)	General inflammation marker	60–75	60–70	0.65–0.75	Primary care; general wards

Abbreviations: ED (Emergency Department), ICU (Intensive Care Unit). AUROC (Area Under the Receiver Operating Characteristic Curve).

**Table 2:** Comparative diagnostic performance of key sepsis biomarkers.

Biomarker	Marker Type	Sens. (%)	Spec. (%)	AU-ROC	Clinical Setting	Remarks	Refs
Procalcitonin (PCT)	Bacterial infection marker; inflammatory mediator	70–77	79–87	0.82–0.86	Emergency department (ED); ICU; antibiotic stewardship pathways.	Most validated biomarker; useful for bacterial vs. non-infectious differentiation and stewardship algorithms.	[24,26,27,28]
Presepsin (sCD14-ST)	Monocyte activation marker	77–86	73–78	0.79–0.88	Early ED triage; acute medical admissions; settings with renal function monitoring.	Comparable to PCT; rises earlier; may improve early diagnosis when combined with clinical scores.	[25]
Neutrophil CD64	Neutrophil activation receptor	87	90	0.94	Adult ICU; Pediatric ICU; high-acuity wards.	Higher diagnostic accuracy than PCT/CRP; useful in ICU settings and for bacterial sepsis detection.	[27]
C-Reactive Protein (CRP)	General inflammation marker	60–75	60–70	0.65–0.75	Primary care; general wards; resource-limited settings.	Widely available but nonspecific; best used in combination with clinical or molecular markers.	[25,28]

Abbreviations: Sens., Sensibility; Spec., Specificity; AU-ROC, area under the receiver operating characteristic curve.

## Data Availability

No new data were created or analyzed in this study. Data sharing is not applicable to this article.
